# A Time-of-Flight Estimation Method for Acoustic Ranging and Thermometry Based on Digital Lock-In Filtering

**DOI:** 10.3390/s22155519

**Published:** 2022-07-24

**Authors:** Qi Liu, Bin Zhou, Jianyong Zhang, Ruixue Cheng, Xuhao Zhao, Rong Zhao, Minglu Dai, Bubin Wang, Yihong Wang

**Affiliations:** 1 School of Energy and Environment, Southeast University, Nanjing 210096, China; liuqi667@seu.edu.cn (Q.L.); seuzxh@seu.edu.cn (X.Z.); zhaorong@seu.edu.cn (R.Z.); danilo@seu.edu.cn (M.D.); wbb@seu.edu.cn (B.W.); wyh@seu.edu.cn (Y.W.); 2 School of Computing, Engineering and Digital Technologies, Teesside University, Middlesbrough TS1 3BX, UK; j.zhang@tees.ac.uk (J.Z.); r.cheng@tees.ac.uk (R.C.)

**Keywords:** acoustic ranging, acoustic thermometry, digital lock-in filtering, electrical conduit, time-of-flight estimation

## Abstract

Accurate ranging and real-time temperature monitoring are essential for metrology and safety in electrical conduit applications. This paper proposes an acoustic time-of-flight (TOF) estimation method based on the digital lock-in filtering (DLF) technique for conduit ranging and thermometry. The method establishes the relationship between the frequency and the time domain by applying a linear frequency modulated Chirp signal as the sound source and using the DLF technique to extract the first harmonic of the characteristic frequencies of the transmitted and received signals. Acoustic TOF estimation in the conduit is then achieved by calculating the mathematical expectation of the time difference between each characteristic frequency in the time-frequency relationship of the two signals. The experimental results with enhanced noise interference on different conduit lengths and various temperature conditions, proved that the proposed DLF method can establish a robust linear time-frequency relationship according to the characteristics of the Chirp signal, and the measurement accuracy of TOF has also been confirmed. Compared to the conventional method, the DLF method provides the lowest absolute error and standard deviation for both distance and temperature measurements with an enhanced robustness.

## 1. Introduction

In conduit construction and the maintenance process in the power industry, the indirect calculation of the conduit length is required. Generally, the conduits buried underground or in complex systems are long and mostly bent. Accurate length measurement and real-time temperature monitoring are challenging and indispensable technical safeguards in conduit laying and maintenance processes.

Traditional contact measurement methods such as scale and thermocouple cannot be applied to lengthy underground conduits. Ranging and thermometry methods based on laser [1,2] and imaging [3,4] technologies are constrained because light can only travel in straight line, so it is unsuitable for lengthy, curved underground pipeline applications. Ultrasonic and microwave ranging [5,6] and thermometry [7,8] methods require considerable power for long-distance measurements due to their shorter wavelengths and faster attenuation, while increased power means an increased size of the sensor, and hence they are inconvenient for such application, particularly for small diameter conduits. The acoustic method has its advantages over the mentioned techniques above.

For convenience, the audio range with an operating frequency falling between 20 Hz and 20 kHz is referred to as acoustic hereinafter in distinction from ultrasound. Different from the laser and camera imaging, when a sound wave propagates in a curved conduit encountering local obstructions such as sludge, pipe bulge, and cable obstacles, the propagation can continue through diffraction. The advantages of the acoustic method also lay in its low cost and high anti-interference natures.

According to the measurement mode, acoustic-based ranging and thermometry are mainly divided into two methods, namely the resonance method [9,10,11] and the pulsed time difference method [12,13]. In the pulsed time difference method [14,15] the length and temperature are measured by estimating the acoustic time-of-flight (TOF) in the conduit, which is the focus of this paper.

In the acoustic TOF estimation, the cross-correlation algorithm is widely used, in which the time delay is computed by identifying the similarity of transmitted and received signals. Based on the direct cross-correlation (DCC) algorithm [16], there have been various transformations and developments. The generalized cross-correlation (GCC), weighted cross-correlation [17], and phase-corrected cross-correlation [18] are such examples to mention. However, these algorithms have a common requirement of wide signal bandwidth, because it is inversely related to the measurement error for TOF. Another well-known method is the least mean-square time delay estimation (LMSTDE) [19,20], in which an adaptive FIR filter is used to model the time difference and interpolate the filter weights to obtain the time delay. Many adjustments and deformations [21,22] have been made to LMSTDE in order to reduce the effect of noisy input caused by the limited filter length, however an accurate TOF could still not be achievable under a low signal-to-noise ratio (SNR) if a small number of filter taps are used. The higher-order statistical time-delay estimation algorithm [23,24] has an advantage to suppress the smoothly correlated Gaussian white noise and extract the signal amplitude and phase information by extending the higher-order spectrum of the multidimensional Fourier transform [25]. However, to achieve a reliable accuracy, strict requirements on the sampling length and resolution of the A/D converter must be satisfied. For other techniques such as the over-zero detection method [26,27], the amplitude squared coherence function method [28], the phase spectrum estimation method [29,30], and the adaptive TOF estimation method [31], although they have been found various applications, none of them are suitable for conduit length measurement and temperature monitoring in relatively harsh field conditions due to inferior and unreliable measurement results at low SNR, limitations on sampling period and accuracy, and high computational load.

Conduits for field applications are generally buried underground or in a complex system close to a production or auxiliary equipment, transportation line or building site. Hence a variety of noise and vibration interferences in a conduit can be expected. Moreover, the bending of a conduit, the obstruction of internal cables, and the sand and dirt mixture left inside during construction pose severe challenges to the acoustic measurement method. It is critical for the acoustic method [32] to be able to suppress noise and overcome physical obstacle interference while ensuring the accuracy and precision of acoustic TOF estimation.

This paper proposes a novel acoustic TOF estimation method based on a digital lock-in filter (DLF) to improve the accuracy of electrical conduit length measurement and temperature monitoring. A linear frequency modulated Chirp signal as the acoustic source is used and the relationship between the first harmonic of the multi-frequency component and the Chirp signal moment is established through digital phase-locking and low-pass filtering of the signal. The acoustic TOF estimation in the conduit is achieved by calculating the mathematical expectation of the time difference between the received and transmitted signals at each characteristic frequency. The accuracy of the DLF method for conduit length and temperature monitoring is verified experimentally by comparison with the classical GCC and GCC-SCOT algorithms. The robustness of the method is tested at various levels of noise intensity with different time spans. The results confirm that both the accuracy and robustness are improved and satisfactory.

## 2. Principle of Acoustic Ranging and Thermometry

**Figure 1:**
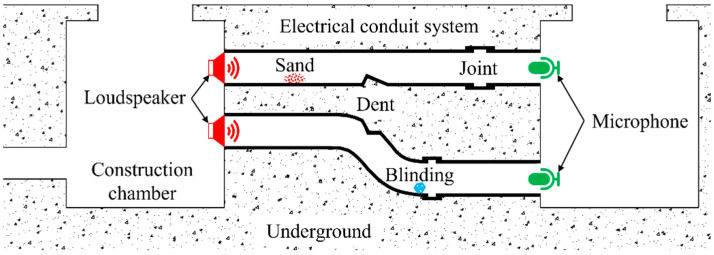
Schematic of the acoustic ranging and thermometry method for electric conduits.

In an electrical conduit system shown in Figure 1, although there are some stones, sand, dents, and joints in the conduit, the sound wave will be transmitted from the loudspeaker (the transmitter) at one end to the microphone (the receiver) fixed at the other end of the conduit through the gas medium due to continuity of the gas in the conduit. The length and temperature of the conduit can be found indirectly by estimating the TOF between the transmitted and received signals at the source and at the receiver by calculation of the time delay algorithm.

Since there is little variation in the enclosed conduit’s electromagnetic, gas state, and composition, the acoustic wave can be assumed to be a linear propagation process. As expressed in Equation (1), in the pre-construction stage, the conduit length *L* is calculated at ambient temperature through the product of the sound speed *c* and the TOF *τ*. Once the construction of the conduit is completed, online measurement of conduit temperature [33,34] can be achieved based on the calibrated conduit length.

(1) $$L=\tau c=\tau \sqrt{\frac{\gamma R}{m}T}$$

where *γ* is the isentropic exponent of the medium, *R* is the universal gas constant, *m* is the molar mass, and *T* is the gas temperature inside the conduit.

It can be found from Equation (1) that the accurate estimation of the acoustic TOF is the priority either for both distance calculating and temperature monitoring. Since the proposed DLF and other time delay estimation methods have already been elaborated elsewhere with the acoustic source parameters, only the sound source signal model will be introduced in Section 2.1.

### 2.1. Acoustic Source Signal Model

The signal model with linear variation in frequency [33,34] has been proven to differentiate signal better from noise hence leading to improved SNR. In this research, the sound source signal is modulated with the widely used linear frequency Chirp. Assuming the presence of additive noise in both the transmitted and received signals of the loudspeaker:

(2) $$\left\{ \begin{array}{l} x_{1}(t)=s(t)+\mu_{1}(t)\\ x_{2}(t)=\xi s(t-\tau )+\mu_{2}(t) \end{array}\right.$$

where *s*(*t*) is the acoustic source signal. *μ*_1_(*t*) and *μ*_2_(*t*) are the random noises contained in the signals. *ξ* is the attenuation coefficient of the acoustic signal. *τ* denotes the TOF between the two signals. *s*(*t*), *μ*_1_(*t*), and *μ*_2_(*t*) are assumed to be uncorrelated.

The transmitted signal *x*_1_(*t*) is:

(3) $$x_{1}(t)=A\cos(2\pi f_{\mathrm{Linear}}t+\Delta \varphi )+\mu_{1}(t)\;\;\;t\in [0,\tau_{s}]$$

where *A* is the signal amplitude, *τ*_s_ is the pulse width, Δ*φ* is the signal phase. *f*_Linear_ denotes the sweeping frequency of the signal, which is expressed as:

(4) $$f_{\mathrm{Linear}}=f_{0}+\frac{B_{w}}{\tau_{s}}t\;\;\;t\in [0,\tau_{s}]$$

where *f*_0_ is the starting frequency at *t* = 0, *B_w_* is the bandwidth in hertz.

**Figure 2:**
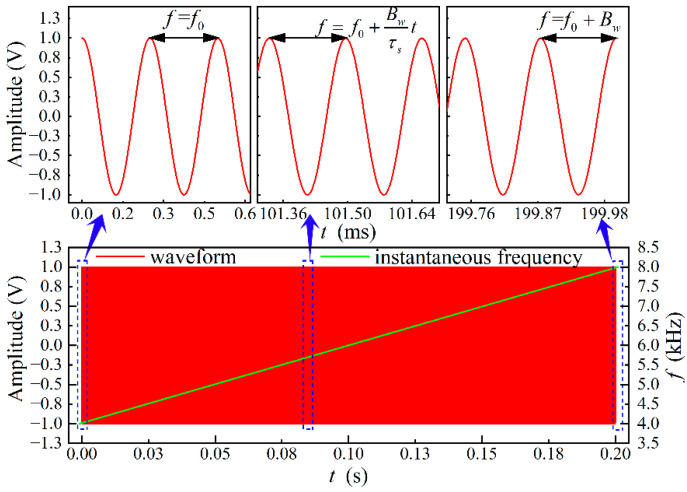
The source signal and its instantaneous frequency.

In order to avoid overlap of the source signal with the low-frequency noise in the field and in line with the best frequency response of the loudspeaker, in this research, the starting frequency and bandwidth of the source signal are set to 4 kHz. The frequency change with time is shown in Figure 2, where the amplitude *A* of the sound source signal is 1 V, and the waveform is a continuously varying cosine from 0 s to 0.2 s. The instantaneous frequency of the signal increases linearly with the time. The frequency varies linearly from 4 kHz to 8 kHz in 0.2 s.

### 2.2. The DLF-Based Acoustic Time-of-Flight Estimation Method

The digital lock-in filtering technique is a versatile signal processing method with a wide range of applications in spectroscopy [2], electricity [35], and magnetism [36] to extract signals at specific frequencies from harsh interference environments. Based on the DLF technique, this paper proposes a novel method that can be used to accurately estimate the TOF.

Once the acoustic signal propagates through the conduit, its waveform may be distorted and attenuated. The intrinsic mode decomposition [37,38] of the acoustic signal reveals that its frequency characteristics generally remain unchanged. By analyzing the instantaneous frequency of the received signal, a new time-frequency relationship can be derived, from which, it can be seen that the offset between the two time-frequency relationships is the acoustic TOF. Traditional frequency domain analysis methods are constrained by the Fourier transform characteristics so an accurate relationship between the time and frequency domains cannot be established. In this paper, digital lock-in filtering techniques are adopted to extract the instantaneous frequencies of the transmitted and received signals, respectively, which can better characterize the local features of the two acoustic signals at different moments.

**Figure 3:**
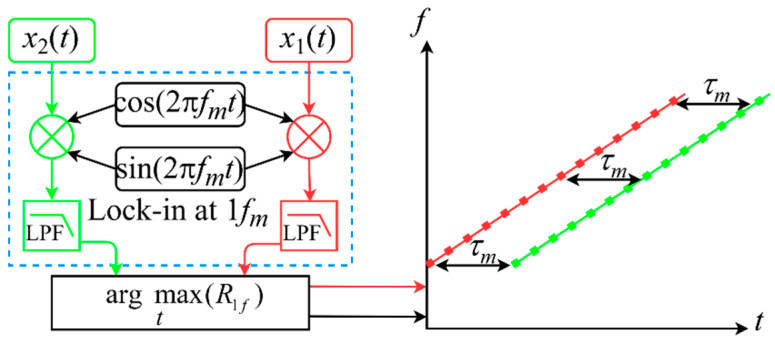
Flow chart of the DLF method for establishing time-frequency relationships.

The DLF method can be used to demodulate the amplitude and phase of each component of the same frequency in the received and transmitted signals by means of the phase-sensitive detection principle. The proposed TOF estimation method uses digital phase-locking and low-pass filtering to extract the first harmonics of the different characteristic frequencies of the acoustic signal. It demodulates the moment of occurrence at that frequency by detecting the peak of the first harmonic, thus establishing the time-frequency relationship of multiple characteristic frequencies. The procedure for calculating the time difference at each characteristic frequency is shown in Figure 3.

In the DLF method, the reference signal *Z*_1_(*t*) and its orthogonal signal *Z*_2_(*t*) with 90° phase shift are assumed to be:

(5) $$\left\{ \begin{array}{l} Z_{1}(t)=B\sin\left(2\pi f_{m}t\right)\\ Z_{2}(t)=B\cos\left(2\pi f_{m}t\right) \end{array}\right.$$

where *B* is the amplitude of the reference and orthogonal signals with characteristic frequency *f_m_* ranging from *f*_0_ to *B_w_* + *f*_0_. Since the first harmonic detection range is determined from the transmitted signal, the expressions for the reference and orthogonal signals are known.

In the DLF method, first the acoustic signal with the reference phase is multiplied by the orthogonal signals to realize the function of phase-sensitive detection. For illustrative purposes, the following is an example of the transmitted signal *x*_1_(*t*), which goes through the multiplier as follows:

(6) $$X_{1f}(t)=x_{1}(t)*Z_{2}(t)=\frac{AB}{2}[\cos(2\pi f_{\mathrm{Linear}}t+2\pi f_{m}t+\Delta \varphi )+\cos(2\pi f_{\mathrm{Linear}}t-2\pi f_{m}t+\Delta \varphi )]+B\cos(2\pi f_{m}t)\mu_{1}(t)$$

(7) $$Y_{1f}(t)=x_{1}(t)*Z_{1}(t)=\frac{AB}{2}[\sin(2\pi f_{\mathrm{Linear}}t+2\pi f_{m}t+\Delta \varphi )-\sin(2\pi f_{\mathrm{Linear}}t-2\pi f_{m}t+\Delta \varphi )]+B\sin(2\pi f_{m}t)\mu_{1}(t)$$

When the characteristic frequencies of the reference and orthogonal signals are the same as the signal to be detected, i.e., *f*_Linear_ = *f_m_*, Equations (6) and (7) can be simplified as:

(8) $$X_{1f}(t)=\frac{AB}{2}\left[\cos(2\pi f_{m}t+\Delta \varphi )+\cos(\Delta \varphi )\right]+B\cos(2\pi f_{m}t)\mu_{1}(t)$$

(9) $$Y_{1f}(t)=\frac{AB}{2}\left[\sin(2\pi f_{m}t+\Delta \varphi )-\sin(\Delta \varphi )\right]+B\sin(2\pi f_{m}t)\mu_{1}(t)$$

After being low-pass filtered, the output of the two signals is obtained as:

(10) $$X_{1f}(t)=\frac{AB}{2}\cos(\Delta \varphi )$$

(11) $$Y_{1f}(t)=\frac{AB}{2}\sin(\Delta \varphi )$$

Therefore, the magnitude of the first harmonic *R*_1*f*_ can be calculated as:

(12) $$R_{1f}=\sqrt{X^{2}{}_{1f}+Y^{2}{}_{1f}}$$

The moment *t* corresponding to the characteristic frequency *f_m_* is obtained from the peak detection of the first harmonic:

(13) $$t_{fm}=\underset{t}{\arg\max}(R_{1f})$$

Therefore, the time delay for each frequency *f_m_* is expressed as:

(14) $$\tau_{m}=t_{\mathrm{received}\_f_{m}}-t_{\mathrm{transmitted}\_f_{m}}$$

where *t*_received_*fm*_ and *t*_transmitted_*fm*_ are the moment of the received and transmitted signals at the *m*th frequency *f_m_*, respectively.

The *m* time-frequency relationships *t_fm_* are obtained by detecting the time differences at all *m* number of characteristic frequencies. TOF can then be obtained by calculating the mathematical expectation of the time difference between the received and transmitted signals over the passband *B_w_*.

(15) $$\tau =E(\tau_{m})=\frac{1}{B_{w}}\sum_{f_{m}=f_{0}}^{f_{0}+B_{w}}\left(\tau_{m}\right)$$

The Chirp signal frequency varies linearly over the pulse width. The analysis of the dynamics of the received acoustic signals by means of nonlinear dynamics metric [39,40] shows that if the loudspeaker’s response is inadequate, this can result in intermodulation distortion of the output acoustic signal. The signal conditioner also causes harmonic distortion of the signal after it has been received by the microphone, leading to a non-linear [41] relationship between frequency and time of the acoustic signal. Due to the known time-frequency characteristics of this pair of acoustic signals, the relationship is fitted linearly by means of a least-squared-based regression method in order to minimize the effects of non-linearity. The linear nature of the Chirp signal is combined to improve the fitting accuracy of the time-frequency relationship by minimizing the sum of squares of the errors. Assume that the linear equation of the time-frequency relationship is:

(16) $$f_{i}=b+kt_{i}+\varepsilon$$

where *b* and *k* are the intercept and slope, respectively, and *ε* is the residual between the actual and the fitting frequency values. The residual sum of squared error Δ*f*(*b*,*k*) can be determined as follows:

(17) $$\Delta f(b,k)=\sum_{i=1}^{n}\varepsilon^{2}=\sum_{i=1}^{n}{\left(f_{i}-b-kt_{i}\right)}^{2}$$

where *f_i_* is the actual value. *b* + *kt_i_* is the targeting value. By making the partial derivatives of the residual sum Δ*f*(*b*,*k*) relative to *b* and *k* zeros, the optimized *b* and *k* can be identified to achieve the minimum Δ*f*(*b*,*k*).

**Figure 4:**
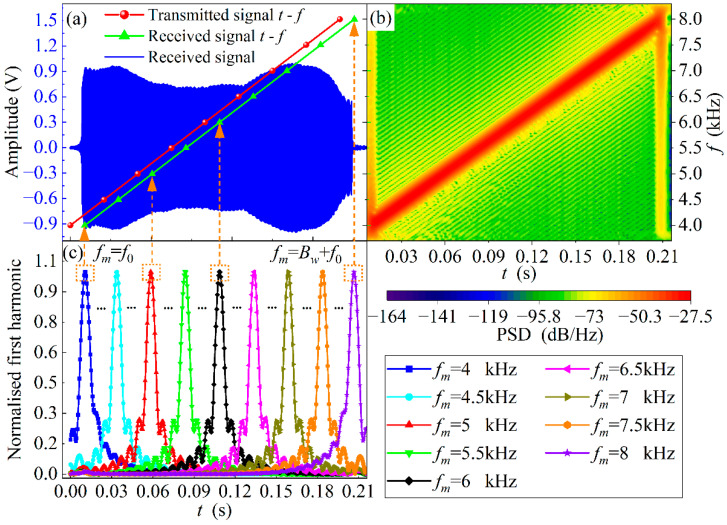
Example of TOF estimation process with DLF method. (**a**) The time-domain waveform of the received signal and time-frequency relationships of transmitted and received signals. (**b**) The acoustic spectrogram of the received signal. (**c**) Normalized first harmonic shown at 0.5 kHz intervals within the signal bandwidth from the starting frequency.

In order to present the process of estimating TOF with the DLF method, an example of an acoustic signal sampled at a conduit length of 3 m is shown in Figure 4. The signal sampling rate is 200 kS/s, the sampling period is 0.22 s, and the starting frequency and bandwidth of the acoustic source signal are 4 kHz.

Figure 4a shows that the received acoustic signal is distorted in waveform amplitude compared to the source signal in Figure 2 after propagating 3 m inside the conduit. The effective pulse width is 0.2 s, which is consistent with the acoustic source. The time lag at the front of the waveform is the acoustic TOF. Corresponding to Figure 4a, the acoustic spectrum of the received signal is depicted in Figure 4b. The acoustic spectrogram is essentially a short-time Fourier transform, which does not provide for both time and frequency resolution of the analyzed signal, but the overall trend of the signal time-frequency correspondence can be clearly seen from this figure. Shown in Figure 4a, the frequency of the received signal varies linearly from 4 kHz to 8 kHz with time over the pulse width, which is the same as the frequency-time relationship of the source signal, except that there is a significant hysteresis in between. The multiple characteristic frequencies of the received signal are digitally phase-locked and filtered. Figure 4c shows the normalized first harmonics at 0.5 kHz intervals from the starting frequency. The peak of the first harmonic coincides with the moment at which that frequency is located. Hence, it can be concluded that a discrete sequence of time-frequency relationships can be derived by detecting the peaks of multiple first harmonics, and the linear interpolation can be assumed between discrete points. The time-frequency relationship of the received signal in Figure 4a is determined with the least-squares-based regression fitting method detailed with Equations (16) and (17).

Similarly, the time-frequency relationship of the transmitted signal is also shown in Figure 4a. Thus, each eigenfrequency corresponds to a time delay as described in Equation (14). The TOF of the acoustic wave can be estimated based on the mathematical expectation of the time delays corresponding to the multiple eigenfrequencies.

### 2.3. Cross-Correlation Time-of-Flight Estimation Method

The cross-correlation algorithm is widely used in the study of acoustic TOF estimation. The operation result reflects the similarity strength of the two signals, and the time delay of the two signals is detected when the similarity is most potent, where the peak position of the cross-correlation function occurs.

Compared to the traditional DCC algorithm, the generalized cross-correlation (GCC) is more flexible in its operation [17]. The cross-power spectrum is obtained by performing a Fourier transform on the two time-domain acoustic signals and, if necessary, pre-processing the cross-power spectrum in the frequency domain by weighting. The inverse Fourier transform is then applied to the cross-power spectrum. The process is as follows:

(18) $$G_{x_{1}x_{2}}(\omega )=F\left[x_{1}(t)\right]F^{*}\left[x_{2}(t)\right]$$

(19) $$R_{x_{1}x_{2}}(\tau )=F^{-1}\left[G_{x_{1}x_{2}}(\omega )\right]$$

where *G_x_*_1*x*2_(*ω*) is the cross-power spectrum of the two signals; *F*[·] stands for Fourier transform; * denotes the complex conjugate; *R_x_*_1*x*2_(*τ*) is the GCC function; *F*^−1^[·] represents inverse Fourier transform function.

The GCC algorithm is whitened and pre-weighted in the frequency domain to enhance the suppression of noise interference. The weighting process of the Smoothed Coherent Transform (SCOT) [42] has a significant suppression effect on noise and is widely used for accurate time delay estimation. The SCOT weighting function is essentially an improvement on the ROTH weighting function [17], which is expressed as follows:

(20) $$\psi_{x_{1}x_{2}}{\left(\omega \right)}_{\mathrm{SCOT}}={\left[G_{x_{1}x_{1}}(\omega )G_{x_{2}x_{2}}(\omega )\right]}^{-1/2}$$

where *G_x_*_1*x*1_(*ω*) and *G_x_*_2*x*2_(*ω*) are the self-power spectra of transmitted and received signals, respectively. Therefore, the GCC-SCOT function is defined as:

(21) $$R_{x_{1}x_{2}}^{\mathrm{SCOT}}(\tau )=F^{-1}\left[\psi_{x_{1}x_{2}}{\left(\omega \right)}_{\mathrm{SCOT}}G_{x_{1}x_{2}}(\omega )\right]$$

Described with Equation (22), in GCC, the accurate estimation of the acoustic TOF is determined by detecting the peak position of the cross-correlation function, and so is in the GCC-SCOT.

(22) $$\tau =\underset{\tau }{\arg\max}[R_{x_{1}x_{2}}(\tau )]$$

## 3. Results Analysis and Discussion

Following the sequence of electrical conduit construction and testing requirements, the calibration measurements of geometric distances were first carried out for different electrical conduits. A laser distance meter (LDM) was used to measure the length of the electrical conduit chosen for the experiments in its straightened state. The measurement was repeated several times to determine the reference standard. The same conduit was measured with the acoustic waveform when the conduit was bent to verify the applicability and feasibility of the proposed DLF method. The experimental study of continuous online monitoring of the conduit temperature was also carried out under disturbing conditions of different noise energy levels, and with mud and gravel inside at different temperatures, by doing so, to validate the method in practical application environments. As a comparison, the GCC, GCC-SCOT, and DLF methods are employed for calculation simultaneously.

### 3.1. Experiment Setup

**Figure 5:**
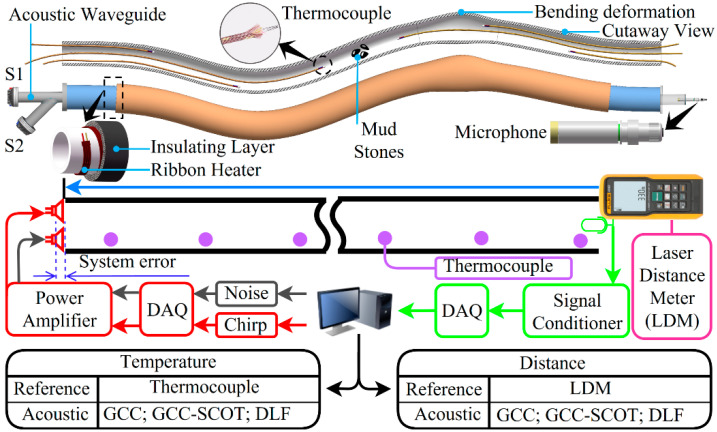
Schematic of the measurement experiment platform.

The experiment measurement system shown in Figure 5 was set up to better imitate the field conditions of the conduit in the laboratory. A linear frequency modulated Chirp signal with a starting frequency and bandwidth of 4 kHz was used as the sound source, sent from the data acquisition card to the loudspeaker via a power amplifier with adjustable gain. At one end of the conduit, the acoustic sound and noise were emitted by Loudspeakers S1 and S2 respectively and transmitted through the conduit. The microphone at the other end was used to receive the signals. The loudspeakers and the microphone were installed utilizing fixed-size acoustic waveguides. The acoustic waveguide installed with S1 and S2 was an internal tee structure. The microphone was 1/2-inch in diameter, with a sensitivity of 50.4 mV/Pa, and a dynamic range of 20 dB to 136 dB, its frequency response range was from 20 Hz to 20 kHz.

The inner diameter of the conduit was 45 mm. A ribbon heater was uniformly wound around the pipe, which was wrapped with insulation material to create different temperature experiment environments. Six type K thermocouples (GG-K-30-SLE made in Shanghai Yaogeng Automation Instrument Co., Shanghai, China) with a diameter of 0.255 mm were evenly spaced 250 mm apart inside the conduit. As can be seen in the cross-sectional view in Figure 5, the thermocouples were wrapped in a breathable glass fiber cloth with a wire diameter of 1 mm in order to avoid direct contact with inner pipe wall to prevent mismeasurement. The gas pressure inside the conduit was kept almost constant when the conduit was closed, and the average value obtained from several thermocouples was used as a reference or standard temperature. The model of the laser distance meter (LDM) was Fluke 404E (Made in Fluke Co., Beijing, China), with a range of 0.2 m to 40 m and measurement precision of ±2.0 mm + 5 × 10^−5^ *L. It was used to calibrate the length of the conduit when it was in the straightened condition. The noise transmitted by S2 was to simulate the noise disturbance from construction, equipment, and traffic in field applications. Also included in the system were: a data acquisition card NI USB6356 (Made in National Instruments Co., Austin, TX, USA) with a sampling rate of 1.25 MS/s and a resolution of 16 bits; a signal conditioner containing phantom power unit and signal amplification circuit.

### 3.2. System Error Calibration

As shown in Figure 5, the distance between the sound output of the loudspeaker and the coil-wrapped sound diaphragm results in a systematic error in the acoustic ranging and thermometry. Since both the measurement system and the loudspeaker structure remain unchanged, this systematic error can be calibrated employing the least-squares method.

(23) $$L_{r}=\varsigma L_{e}+\Delta L$$

where *L_r_* is the reference length measured by the LDM, *L_e_* is the estimated length calculated through the acoustic method, Δ*L* is the system error, and *ς* is a scale factor by reason that the acoustic signal distortion loss due to conduit bends and obstacles in the pipe. In the experiment, the correction factor and system error were 1.0024 and 8.16 mm, respectively, so the correct distance could be calculated.

### 3.3. Analysis of the Measurement Results

#### 3.3.1. Acoustic Ranging and TOF Estimation in Different Conduit Length

Following the construction of the electrical conduits, the different conduit lengths were first measured using three different time-delay estimation algorithms to investigate the accuracy of the proposed DLF method for different TOF. The internal temperatures of the closed conduits with different lengths were kept the same and maintained during the measurements and the six thermocouples arranged at the ends of the conduits were used to indicate the actual temperature. The electrical conduits were cut into 8 different pieces with the lengths of 0.503 m, 1.021 m, 2.032 m, 3.022 m, 4.017 m, 5.023 m, 6.993 m, and 10.022 m, respectively, based on LDM measurements at their straight states. Once the experimental bench and sensors were all installed and arranged, the temperature in the conduit was monitored using thermocouples, and as the experiments for length measurement using acoustic methods were carried out the temperature became stable. The average temperature of the six thermocouples was 26.31 °C when the thermal equilibrium was reached, and the theoretical speed of sound in the conduit was 345.82 m/s.

The length for each of the mentioned conduit above was measured in the pre-construction phase, which was free from noise and vibration interference. The Chirp acoustic waves were transmitted from S1, and received by the microphone to estimate the acoustic TOF across each of eight different conduits over their full lengths. The number of measurements for each conduit was 200.

**Figure 6:**
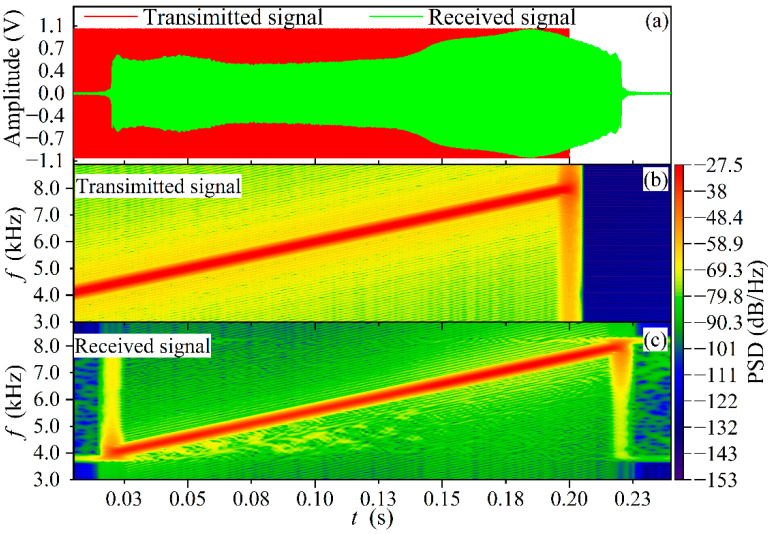
Signal waveform and acoustic spectrum at conduit length of 7 m. (**a**) Transmitted and received acoustic signal waveforms for a conduit length of 7 m. (**b**) Acoustic spectrum of the transmitted signal. (**c**) Acoustic spectrum of the received signal.

In the example of the propagated acoustic signal travelling a conduit of 7 m, it can be seen from Figure 6a that a slight waveform distortion occurred due to the conduit bending, gravel, obstruction of the thermocouple wire, and gas attenuation. A significant time delay of the received signal to the transmitted signal is clearly shown, this time is the TOF to be estimated. From the acoustic spectrum of the two signals in Figure 6b,c, it is evident that the attenuation of the waveforms did not affect the time-frequency relationship of the received signals. This time delay is also obviously reflected in the corresponding acoustic spectrum of the received signal in Figure 6c. This phenomenon provides an explanation of the proposed DLF method.

**Figure 7:**
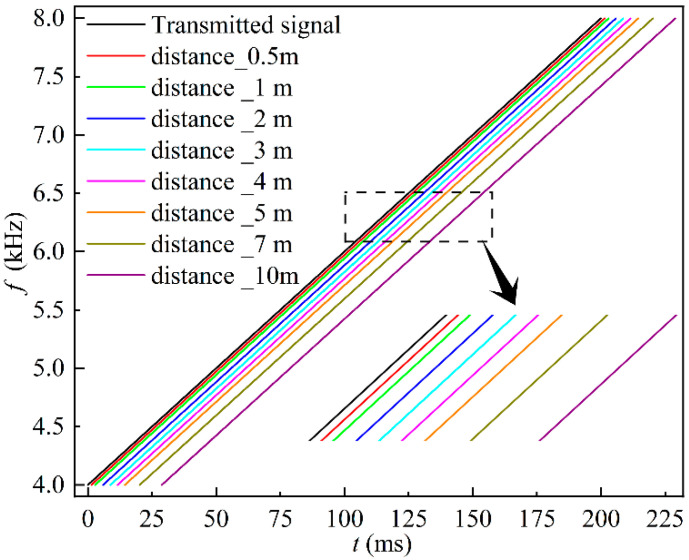
The time-frequency relationships of different lengths of conduits are extracted by the DLF method.

The DLF, GCC, and GCC-SCOT methods were used to process the acoustic signals obtained from the eight conduits with different lengths mentioned above. Figure 7 shows the time-frequency relationship for each conduit at the given length calculated using the DLF method. The time frequency graphs of TOF are parallel for different conduit lengths, indicating the distinct time taken for the acoustic wave travelling a given journey.

**Figure 8:**
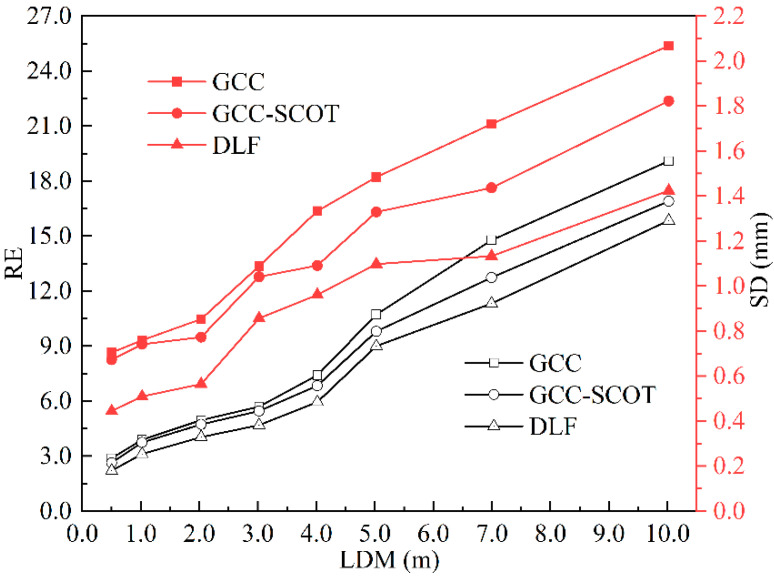
RE and SD results for acoustic methods at different conduit lengths.

Figure 8 provides the details of the analysis, where the measured TOFs were converted to length for the three acoustic methods. Taking the lengths determined with LDM measurements as references, the relative error (RE) and standard deviation (SD) of the three acoustic methods were compared. The results are depicted in Figure 8. Apparently, the graphs in this figure confirm that both RE and SD for all three different methods increase with distance, and both errors with the GCC-SCOT are slightly smaller than with the GCC over the entire range. The DLF method has the best performance in this regard, the maximum RE under DLF is 6.26%, and 16.99% smaller than that derived with GCC and GCC-SCOT, respectively. The DLF method therefore offers the best measurement accuracy, and the lowest SD also demonstrates the best stability over multiple repeated measurements.

#### 3.3.2. Experiments on Temperature Monitoring with Noise Interference at Different Energy Levels

**Figure 9:**
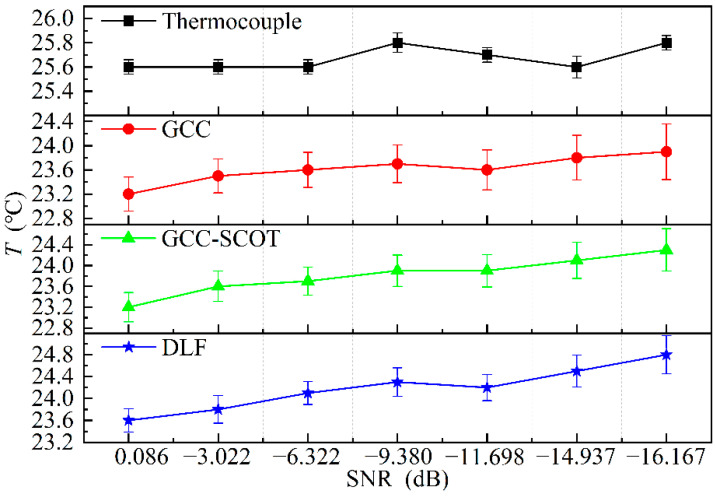
Temperature measurement results in different SNR conditions.

The production or auxiliary equipment, transport, and building construction close to an electrical conduit can generate various noises and vibrations inside, which can interfere with the temperature monitoring by acoustic methods. The vibration effect is not considered in this paper as underground vibrations are mainly low frequency, well below the bandwidth of the signal used for measurement. The previous studies have shown that the noise in underground power conduits is mainly Gaussian white noise [9]. The noise immunity of the DLF method was verified by adding Gaussian white noise to the conduit via S2 and the noise energy level was set by adjusting the power amplifier’s gain. In this section, the thermocouples were evenly aligned in a conduit of 3 m length. The experiments were carried out without heating at seven interfering SNR levels, and the results are shown in Figure 9. The number of measurements at each SNR level was 200.

Due to the closeness of the measured results from the three acoustic methods and the tendency for the error bars to overlap, Figure 9 displays them separately for clarity. The graphs show that the results based on all of the three acoustic thermometry are lower than that given by the thermocouple. The temperature measured by the thermocouple increases by approximately 0.3 °C as the measurement time increases, and the trend is followed by the acoustic measurement results. But the temperatures measured using the DLF method is closest to that obtained using the thermocouple. The maximum absolute error of the DLF is 16.7% and 16.3% lower than that of GCC and GCC-SCOT methods. Thus, it can be concluded that DLF provided the best measurement accuracy.

#### 3.3.3. Experiments on Temperature Monitoring at Different Temperatures

**Figure 10:**
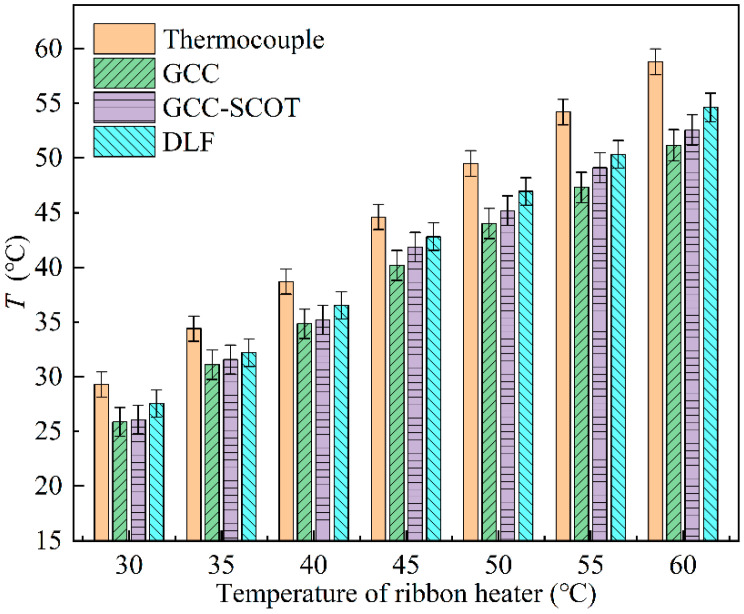
Temperature results of the four methods in different temperature conditions.

The use of cables in electrical conduits is accompanied by heat generation, which can be dangerous when too much heat builds up. In the experiments, the temperature of the ribbon heater was adjusted utilizing a temperature controller to produce different temperature conditions. The temperature conditions are shown in Figure 10, each of which was measured after the temperature controller had been adjusted and a steady temperature reading was reached. The noise was added in the experiments to simulate interference signal. The SNR of the experiment environment was approximately equal to −9.380 dB, and the number of measurements per temperature condition was 200.

Since the upper limit of the temperature controller is 65 °C, seven different temperature conditions were set up ranging from 30 °C up to 60 °C. Figure 10 shows that the thermocouple readings are higher than that of acoustic methods and have small spread (indicated by shorter error bars), probably due to the fact that the glass fibers wrapped around the thermocouple were in contact with the inner conduit wall for a long time, resulting in the temperature being closer to the wall by heat transfer while the acoustic method measured the gas temperature inside the conduit and therefore gave lower results than the thermocouple. The DLF temperature results were higher than the GCC and GCC-SCOT methods with maximum temperature differences of 6.8% and 5.7% times higher and were closest to the thermocouple measurements. The GCC-SCOT results were better than the GCC method in terms of temperature and error. The DLF method had the lowest error, with maximum errors of 9.5% and 7.2% lower than the GCC and GCC-SCOT methods, respectively, and had the best measurement stability.

## 4. Conclusions

This paper proposes a new acoustic time-of-flight estimation method to achieve high accuracy in ranging and thermometry systems. The proposed DLF method is more interference immune which can extract the first harmonics of time-domain signals of low SNR from various noise and vibration disturbing environments. The relationship between time and frequency signals is established by harmonic peak detection. As both the transmitted and received signals have highly similar time-frequency characteristics, an accurate estimation of the acoustic TOF is required that can be attained by solving for the mathematical expectation of the time difference between the two signals so that the conduit length and the temperature can be calculated.

In order to simulate more closely to the on-site conduit conditions, an experiment platform and system for length and temperature measurement were constructed in the laboratory. The signal interference encountered in the field measurement due to physical disturbances from sand, gravel, and mud in the conduit, as well as caused by nearby construction and traffic, and different temperature environments was assumed to be Gaussian white noise. Compared with the conventional TOF estimation methods GCC and GCC-SCOT, the proposed DLF method can give more accurate measurement for conduit length and temperature where the temperature may fluctuate. The DLF method has the advantages of an extensive measurement range, higher accuracy, and robustness so it is more suitable for field applications.

## Data Availability

Not applicable.
